# Digital exclusion and functional dependence in older people: Findings from five longitudinal cohort studies

**DOI:** 10.1016/j.eclinm.2022.101708

**Published:** 2022-10-31

**Authors:** Xinran Lu, Yao Yao, Yinzi Jin

**Affiliations:** a Department of Global Health, School of Public Health, Peking University, Beijing, China; b China Center for Health Development Studies, Peking University, Beijing, China; c Institute for Global Health and Development, Peking University, Beijing, China

**Keywords:** Digital exclusion, Basic activities of daily living, Instrumental activities of daily living, Functional dependency, Older adults

## Abstract

**Background:**

Older people are more likely to be excluded from the digital world, and this has been linked to poor health outcomes. The extent and direction of the influence of digital exclusion on functional dependency is, however, not well understood. We aimed to investigate the association between digital exclusion and functional dependency among older adults from high-income countries (HICs) and low- and middle-income countries (LMICs).

**Methods:**

In this multicohort study, we pooled individual-level data from five longitudinal cohort studies that included nationally representative samples of older adults across 23 countries, including the Health and Retirement Study (HRS), the English Longitudinal Study of Aging (ELSA), the Survey of Health, Ageing and Retirement in Europe (SHARE), the China Health and Retirement Longitudinal Study (CHARLS), and the Mexican Health and Aging Study (MHAS). The digital exclusion was recorded as an absence from internet use by self-reported. We assessed basic activities of daily living (BADL) and instrumental activities of daily living (IADL), and we used interval-of-need methods to categorize the functional dependency. We applied generalized estimating equations models fitting Poisson model to investigate the association of digital exclusion with difficulties in BADL or IADL and functional dependency, adjusting for the causal-directed-acyclic-graph (DAG) minimal sufficient adjustment set (MSAS), including gender, age level, labour force status, education, household wealth level, marital status, and co-residence with children.

**Findings:**

We included 108,621 participants recruited between 2010 and 2018 with a median follow-up of 3 phrases. Digital exclusion in older adults varied across countries, ranging from 23.8% in Denmark (SHARE) to 96.9% in China (CHARLS). According to the crude model, digital exclusion was significantly associated with functional dependency. In the MSAS-adjusted model, those associations remained statistically significant: HRS (incidence rate ratio [IRR] = 1.40, 95% confidence interval [CI] 1.34–1.48 for BADL; 1.71 [1.61–1.82] for IADL), ELSA (1.31 [1.22–1.40] in BADL and 1.37 [1.28–1.46] in IADL), SHARE (1.69 [1.61–1.78] in BADL and 1.70 [1.63–1.78] in IADL), CHARLS (2.15 [1.73–2.67] in BADL and 2.59 [2.06–3.25] in IADL), and MHAS (1.15 [1.09–1.21] in BADL and 1.17 [1.09–1.25] in IADL). In the subgroup analyses, the associations were more pronounced in the oldest-old (aged ≥ 80 years old).

**Interpretation:**

There is a substantial proportion of older adults who are excluded from the Internet, especially those in LMIC. Older people excluded from the Internet regardless of whether they live in HICs or LMICs are more likely to develop functional dependency. It should be made a priority to remove barriers to Internet access in order to assist older people in maintaining their independence and, consequently, to reduce the care burden associated with the ageing population worldwide.

**Funding:**

The National Natural Science Foundation of China (No. 71904004).

**Research in context**

**Evidence before this study**

We searched PubMed, Web of Science, SCOPUS, PsycINFO, and the Cochrane Library using the keywords “digital exclusion”, “digital inclusion”, “digital divide”, “internet access”, “internet use”, “activities of daily living”, “functional dependence”, “disability”, “aging/ageing”, and “older adults” with no date restrictions. Up to August 2022, we yielded four relevant studies. Two cross-sectional studies conducted in Brazil and Japan determined that the frequent use of the internet has been associated with better performance on basic activities of daily living (BADL) and instrumental activities of daily living (IADL), yet a cross-sectional study in China shows no association between internet use and ADL-defined disability. In a longitudinal study from England, digital literacy was associated with a lower prevalence of IADL impairment. However, little research has been conducted on the prevalence of digital exclusion among older people from a global perspective. Further, the direction and the extent to which digital exclusion influences functional dependency in late life has been largely understudied, with limited study from LMICs. Therefore, we aimed to examine the association between digital exclusion and functional dependency among older adults from both HICs and LMICs.

**Added value of this study**

Since growing concerns have been raised about the impact of the digital divide on health since the COVID-19 pandemic, the present study examined digital exclusion among older adults, a vulnerable group that has been understudied. To enhance the generalisation, the present study included five comparative cohort studies (HRS, ELSA, SHARE, CHARLS and MHAS) representing 108,621 participants from 23 countries, including both HICs and LMICs. As far as we know, it is the first cross-cultural and longitudinal study to demonstrate an association between digital exclusion and functional dependence among older adults. We further found that the associations were stronger in the oldest-old (≥80 years).

**Implications of all the available evidence**

The present study provides evidence that older people are more likely to face digital exclusion in LMICs than in HICs. Older adults would face higher risk of dependency and need more care if they were excluded from the Internet. The implementation of well-designed and tailored digital inclusion strategies will likely support healthy ageing by preventing functional dependence in older populations and decreasing health disparities relevant to the digital divide across LMICs as well as HICs.

## Introduction

As the population ages worldwide, functional dependence is on the rise, which means that people in older age require physical assistance due to degenerated body functions, placing substantial medical and care burdens on families and society. Maintaining functioning independence in later life has become a public health priority.^1^ Digital exclusion, defined as the inequity in access and capability to use Information and Communications Technologies (ICTs) such as the internet,^2^ has been associated with poor physical and psychological health status in older people.^3^, ^4^, ^5^ However, the association between digital exclusion and functional dependency in older adults, particularly in low- and middle-income countries (LMICs), has been largely understudied.

Worldwide, older people constitute the largest proportion of non-users of the internet, and the pattern of internet use is similar when looking at digital skills.^6^ Older adults are particularly excluded from digital services due to barriers to using ICTs - the refusal to use the internet, inability to afford internet access or ICT devices, or a lack of literacy and skills to utilize the internet.^2^^,^^7^ Excluding older people from the digital world is part of social exclusion, which has been associated with physical impairments, such as frailty and functional declines.^8^^,^^9^ Digital exclusion prevents individuals from seeking health information online and receiving medical services remotely, which may lead to exacerbated health disparity and poorer health outcomes among older adults.^10^ It was reported that digital exclusion is associated with elevated health care needs and a lower risk of impairment in instrumental activities of daily living.^5^^,^^11^^,^^12^ However, prior studies were either cross-sectional in design or had a relatively small sample size, limiting the generalizability of their findings. In addition, the pattern of association of digital exclusion with functional dependence could be different between LMICs and high-income countries (HICs), given the level of digitalization varied tremendously across the countries.^2^ Further, it remaines to be determined which populations are particularly vulnerable to the relationship between digital exclusion and functional dependency.

To fill these research gaps, we conducted a cross-cultural, longitudinal analysis based on five large, comparative cohort studies between 2010 and 2018 that represented older adults across 23 countries from three continents – North America, Europe, and Asia. In the present study, we investigated the patterns of digital exclusion among participants from five cohorts and associated them with functional dependency. We conducted subgroup analyses to determine which groups of the population were more likely to suffer from digital exclusion in terms of functional dependency.

## Methods

### Study design and participants

Data were obtained from five international cohorts of ageing: Health and Retirement Study (HRS), English Longitudinal Study of Ageing (ELSA), Survey of Health, Ageing and Retirement in Europe (SHARE), China Health and Retirement Longitudinal Study (CHARLS), and Mexican Health and Aging Study (MHAS). The five surveys used here were designed to provide comparable results.^13^, ^14^, ^15^, ^16^, ^17^ They all provide information on digital exclusion and measures on functional dependency of individuals aged 60 and over. In this study, we use data from a similar time range: 2010–16 for HRS, 2010–18 for ELSA, 2013–17 for SHARE, 2011–18 for CHARLS, 2012–18 for MHAS.

We excluded participants who were younger than 60 years of age and those with missing data regarding functioning ability, digital exclusion or covariates. After excluding these participants, 17,149 participants from HRS, 8683 participants from ELSA, 58,848 participants from SHARE, 11,634 participants from CHARLS, and 12,307 participants from MHAS were available for the analysis (Study flow diagram: Supplementary Fig. S1).

### Procedure

The measurements of exposure (digital exclusion), outcome (functional ability and dependency), and covariates (demographics, socio-economic positions, living arrangements, lifestyles, and the presence of chronic conditions as well as mental diseases are repeated in the five cohorts.

Data on digital exclusion were collected through self-completed questionnaires. In HRS, digital exclusion was assessed using a single question: ‘‘Do you regularly use the Internet (or the World Wide Web) for sending and receiving e-mail or for any other purpose, such as making purchases, searching for information, or making travel reservations?’‘. In ELSA, the participants were asked of the frequency they use the internet, and the responses ranged from 1 = ‘‘Every day, or almost every day’’ to 6 = ‘‘never.’’ In SHARE, the independent variable was constructed based on the following question: “In the last 7 days, have you used the Internet at least once for e-mailing, searching for information, making purchases, or for any other purpose?”. For CHARLS, the independent variable was assessed using the question “Have you used the Internet in the past month?”. For MHAS, digital exclusion on an individual level was not available, so the alternative question was asked: “Do you have Internet access at home?”. The response “no” (HRS, SHARE, CHARLS, and MHAS) or a frequency of less than once a week (SHARE) was categorized as digital exclusion, while the response “yes” or a frequency of at least once a week was considered as digital inclusion.

In our study, functional dependency is defined as a participant's inability to perform the basic ADLs and IADLs on their own basic or physical ADLs are those skills required to perform one's daily physical tasks, including dressing, bathing, feeding, transferring from bed to chair, toileting, and maintaining continent. IADLs include more complex activities related to the ability to live independently, which constitute doing housework, cooking, shopping, managing money, and taking medication. The measure of functional ability was based on the performance of basic activities of daily living (BADL) and instrumental activities of daily living (IADL). BADL was measured using the Katz scale with six items including eating, dressing, getting in/out of bed, using the toilet, bathing, and walking.^18^ According to Lawton, IADL includes preparing hot meals, taking medications, managing money, shopping for groceries, cleaning the house, and using the telephone.^19^ Cleaning the house in IADL was not available for HRS and the first wave in CHARLS. For MHAS, both IADL items cleaning the house and using the telephone were not available. Participants were asked if they had any difficulties with those items and the answer “yes” and “no” were coded into 1 and 0, respectively. The sum of the items of BADL and IADL was calculated respectively into a score ranged from 0 to 6. The IADL score in HRS is 5, whereas the IADL in MHAS is 4. The score of more than 0 was defined as difficulties in BADL or IADL and 0 as no difficulties.

To measure functional dependency, we used the so-called interval-of-need methods developed by Isaacs and Neville.^20^ This method, which has been used to forecast the care needs in England,^21^ categorises participants based upon the frequency with which they require care: high dependency (needing care 24/7), medium dependency (needing help several times per day), low dependency (needing care less often than daily), or independent (not needing care) (Table 1).

**Table 1:** Functional dependency by interval-of-need dependency categorisation.

Categories	Definition
High dependency	Difficulty in eating, dressing, getting in/out of bed, using the toilet, or walking
Medium dependency	Difficulty in preparing hot meals or taking medications, and no-difficulty in the items defined in high dependency
Low dependency	Difficulty in bathing, managing money, shopping for groceries, using the telephone, or cleaning the house, and no-difficulty in the items defined in medium and high dependency
Independent	No-difficulty in the items above

Covariates were identified through literature reviews. We included demographics (age level and gender), socio-economic positions (education, labour force status, household wealth level), living arrangements (marital status and co-residence with children), lifestyles (smoking, drinking), and the presence of chronic conditions (ever had hypertension, stroke, cancer), as well as mental symptoms (depressive symptoms, cognitive impairment). Further details of the covariates are available in Supplements.

### Statistical analysis

We described the characteristics of the observations in the HRS, ELSA, SHARE, CHARLS, and MHAS, separately. For descriptive statistics, the mean (SD) or the median (Q1–Q3) was used for continuous variables and numbers and percentages for categorical variables.

To tackle the intercorrelation for repeated measures in each cohort, we performed generalized estimating equations (GEE) models fitting Poisson model with exchangeable correlation and calculated incidence rate ratios (IRRs) and 95% confidence intervals (CIs) with robust sandwich standard error (SE) to investigate the associations of digital exclusion with difficulties in performance of BADL and IADL. We included demographic and socio-economic determinants, living arrangements, lifestyle factors, the presence of chronic diseases, and mental symptoms as the covariates. The minimal sufficient adjustment set (MSAS) was identified using a causal directed acyclic graph (DAG) for estimating the total effect of digital exclusion on functional dependence (Supplementary Fig. S2). We utilized four models in the analyses, of which Model 1 was a crude model. In Model 2, we accounted for age level and gender. Model 3 controlled MSAS, further including labour force status, education, household wealth level, marital status, and co-residence with children based on Model 2. Model 4 was fully adjusted to include all covariates. We used a conditional fixed-effects multinomial logit model with intraindividual-clustered standard error to examine the association of digital exclusion with functional dependency by interval-of-need categorisations. To investigate the potential influence of digital exclusion on specific items of functioning ability, we also examined the association between digital exclusion and performance in each item of BADL/IADL across all the five cohorts by using the same MSAS-controlled model.

We performed subgroup analyses by gender (male/female), age (≥80/<80 years), labour force status (currently not working/currently working without retirement/currently working after retirement), education (less than upper secondary/upper secondary and vocational training/tertiary), household wealth (low tertile/medium tertile/high tertile), marital status (married or partnered/single), co-residence with children (yes/no), and depressive symptoms (yes/no). Forest plots were drawn to visualize the adjusted ORs in sub-populations.

To test the robustness of our findings, we did four sets of sensitivity analyses. Firstly, we repeated the GEE analyses to associate the digital exclusion and scores of BADL and IADL as continuous variables, respectively. Secondly, we repeated the GEE analyses fitting Poisson model in the participants with baseline non-difficulty in BADL and IADL and functional independency and more than one follow-up visit. To reduce the recall bias, we then repeated the analyses of the associations of digital exclusion with difficulties of BADL and IADL by excluding participants with severe cognitive impairment at baseline. Finally, to deal with biases due to dropout, we constructed inverse-probability weights (IPWs) to model the attrition among the cohorts and then fitted the GEE analyses of difficulty in terms of BADL and IADL with these weights. All analyses were performed by Stata 16.0 and 17.0 (StataCorp, College Station, USA). We used “xtgee” command for GEE model, “xtmlogit” command for fixed-effects multinomial logit model, and “xtrccipw” command for GEE analysis with IPW. Two-sided *p* values <0.05 were considered to be statistically significant.

### Role of funding

The study sponsor has no role in study design, data analysis and interpretation of data, the writing of manuscript, or the decision to submit the paper for publication.

### Ethical statement

We utilized de-identified data from public databases, including HRS, ELSA, SHARE, CHARLS and MHAS. The ethical approval was covered by the original surveys and was not necessary for the present study.

## Results

**Fig. 1:**
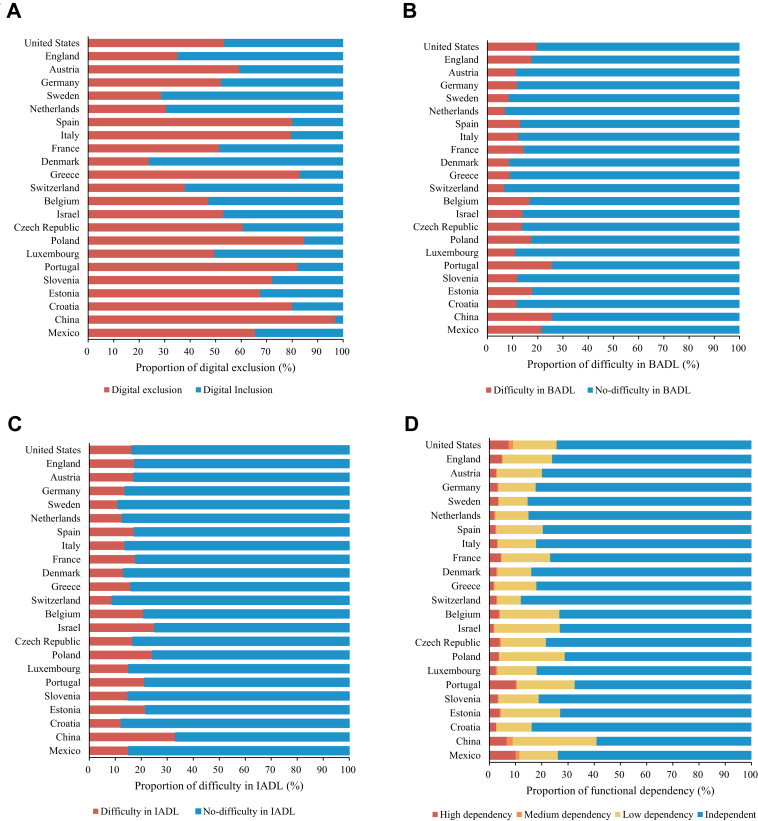
Proportion of digital exclusion, difficulties in BADL, difficulties in IADL, and functional dependency.

The characteristics of observations in the five cohort studies are presented in Table 2. The median ages of included participants for HRS, ELSA, SHARE, CHARLS, and MHAS were about 70, and the male participants ranged from 41.3% in HRS to 48.2% in CHARLS. Participants in the surveys of HRS, ELSA, SHARE, CHARLS, and MHAS were all scored at the median of 0 in terms of BADL/IADL. More than 70% of the participants were independent, except for less than 60% of those CHARLS, according to interval-of-need dependency categorisation. The proportion of digital exclusion in older adults varied widely across countries, ranging from 23.8% in Denmark (SHARE) to 96.9% in China (CHARLS) (Fig. 1). The pooled proportion of digital exclusion in SHARE is 57.4%, while the digital exclusion rate is 53.2% in HRS, 30.4% in ELSA, and 65.5% in MHAS (Supplementary Table S1).

**Table 2:** Descriptive statistics in HRS, ELSA, SHARE, CHARLS, and MHAS.

	HRS (N = 49,583)	ELSA (N = 27,338)	SHARE (N = 96,184)	CHARLS (N = 23,342)	MHAS^a^ (N = 26,968)
Age, median (Q1–Q3)	72 (65–78)	69 (64–76)	70 (12–77)	67 (63–72)	69 (65–76)
Male gender	20,469 (41.3)	12,853 (47.0)	42,160 (43.8)	11,261 (48.2)	11,854 (44.0)
Labour force status					
Currently not working	36,479 (73.6)	21,670 (79.3)	78,390 (81.5)	11,214 (48.0)	19,162 (71.1)
Currently working without retirement	10,404 (21.0)	4846 (17.7)	10,737 (11.2)	11,095 (47.5)	7806 (28.9)
Currently working after retirement	2700 (5.45)	822 (3.01)	7057 (7.34)	1033 (4.43)	
Education					
Less than upper secondary	9381 (18.9)	8003 (29.3)	42,587 (44.3)	21,649 (92.7)	23,949 (88.8)
Upper secondary and vocational training	28,989 (58.5)	13,955 (51.0)	33,529 (34.9)	1363 (5.84)	691 (2.56)
Tertiary	11,213 (22.6)	5380 (19.7)	20,068 (20.9)	330 (1.41)	2328 (8.63)
Household wealth					
Low tertile	14,998 (30.2)	7464 (27.3)	28,993 (30.1)	7253 (31.1)	10,825 (40.1)
Medium tertile	14,974 (30.2)	9423 (34.5)	33,028 (34.3)	9231 (39.5)	7322 (27.2)
High tertile	19,611 (39.6)	10,451 (38.2)	34,163 (35.5)	6858 (29.4)	8821 (32.7)
Married or partnered	29,735 (60.0)	19,171 (70.1)	69,294 (72.0)	18,341 (78.6)	17,000 (63.0)
Co-residence with children	7908 (15.9)	224 (0.82)	13,692 (14.2)	9270 (39.7)	18,302 (67.9)
Smoking	5181 (10.4)	2462 (9.01)	13,667 (14.2)	6294 (27.0)	2689 (9.97)
Alcohol drinking	25,587 (51.6)	23,557 (86.2)	47,555 (49.4)	7245 (31.0)	5905 (21.9)
Ever had hypertension	32,657 (65.9)	12,854 (47.0)	52,886 (55.0)	9456 (40.5)	16,907 (62.7)
Ever had stroke	4957 (10.0)	1341 (4.91)	7320 (7.61)	1365 (5.85)	1192 (4.42)
Ever had cancer	8946 (18.0)	3621 (11.9)	10,595 (11.0)	378 (1.62)	1101 (4.08)
Depressive symptom	10,314 (20.8)	5057 (18.5)	26,091 (27.1)	8931 (38.3)	8760 (32.5)
Cognitive impairment	2275 (4.59)	1047 (3.83)	5117 (5.32)	327 (1.40)	1652 (6.13)
Digital exclusion	26,394 (53.2)	8316 (30.4)	55,201 (57.4)	22,622 (96.9)	17,653 (65.5)
BADL, median (Q1–Q3)	0 (0–0)	0 (0–0)	0 (0–0)	0 (0–1)	0 (0–0)
IADL, median (Q1–Q3)	0 (0–0)	0 (0–0)	0 (0–0)	0 (0–1)	0 (0–0)
Difficulty in BADL	0.42 (1.07)	0.33 (0.90)	0.26 (0.85)	0.54 (1.16)	0.45 (1.10)
Difficulty in IADL	0.30 (0.82)	0.31 (0.81)	0.33 (0.95)	0.72 (1.29)	0.26 (0.73)
Functional Dependency					
Independent	36,869 (74.4)	20,793 (76.1)	76,781 (79.8)	13,792 (59.1)	19,929 (73.9)
Low dependency	8238 (16.6)	5162 (18.9)	16,063 (16.7)	7475 (32.0)	3956 (14.7)
Medium dependency1	841 (1.70)	94 (0.34)	374 (0.39)	529 (2.27)	383 (1.42)
High dependency	3635 (7.33)	1289 (4.72)	2966 (3.08)	1546 (6.62)	2700 (10.0)

BADL: basic activities of daily living; CHARLS: China Health and Retirement Longitudinal Study; ELSA: English Longitudinal Study of Ageing; HRS: Health and Retirement Study; IADL: instrumental activities of daily living; MHAS: Mexican Health and Aging Study; SHARE: Survey of Health, Ageing and Retirement in Europe.

Data are N (%) for categorical variables or mean (SD) or median (Q1-Q3) for continuous variables.

a For MHAS, the question on retirement was unavailable, so labour force status was recoded into currently working and currently not working.

Table 3 shows the association between digital exclusion and difficulties in performing BADL and IADL. In the crude model (Model 1), digital exclusion was significantly associated with difficulties in both BADL and IADL. In the MSAS adjusted model (Model 3), those associations remained statistically significant in HRS (1.40 [1.34–1.48] for BADL; 1.71 [1.61–1.82] for IADL), ELSA (1.31 [1.22–1.40] in BADL and 1.37 [1.28–1.46] in IADL), SHARE (1.69 [1.61–1.78] in BADL and 1.70 [1.63–1.78] in IADL), and MHAS (1.15 [1.09–1.21] in BADL and 1.17 [1.09–1.25] in IADL). The associations are stronger in CHARLS (2.15 [1.73–2.67] in BADL and 2.59 [2.06–3.25] in IADL). All the *p* values above were under 0.001. This statistically significant association will be attenuated in the fully adjusted model (Model 4) among all cohort studies. When categorized functional dependency according to interval-of-need method, older adults excluded from the Internet were more likely to suffer from functional dependency in four cohort studies except for MHAS (Table 4). In HRS, ELSA, SHARE, and CHARLS, the digital exclusion significantly associates with each item of BADL and IADL (*p* values < 0.001). Items such as eating and preparing hot meals were nearly statistically significant in the MHSA (Supplementary Table S2).

**Table 3:** Association between digital exclusion and difficulties in BADL and IADL.

		HRS			ELSA			SHARE			CHARLS			MHAS		
		IRR	95% CI	p value	IRR	95% CI	p value	IRR	95% CI	p value	IRR	95% CI	p value	IRR	95% CI	p value
Difficulty in BADL	Model 1	1.92	(1.83–2.01)	<0.001	1.75	(1.65–1.87)	<0.001	2.61	(2.49–2.72)	<0.001	2.73	(2.23–3.34)	<0.001	1.27	(1.20–1.34)	<0.001
	Model 2	1.78	(1.70–1.87)	<0.001	1.63	(1.53–1.75)	<0.001	2.14	(2.04–2.24)	<0.001	2.55	(2.08–3.14)	<0.001	1.23	(1.17–1.30)	<0.001
	Model 3	1.40	(1.34–1.48)	<0.001	1.31	(1.22–1.40)	<0.001	1.69	(1.61–1.78)	<0.001	2.15	(1.73–2.67)	<0.001	1.15	(1.09–1.21)	<0.001
	Model 4	1.31	(1.24–1.37)	<0.001	1.21	(1.13–1.30)	<0.001	1.43	(1.36–1.51)	<0.001	2.04	(1.63–2.55)	<0.001	1.07	(1.01–1.13)	0.012
Difficulty in IADL	Model 1	2.48	(2.35–2.62)	<0.001	1.95	(1.83–2.08)	<0.001	2.77	(2.66–2.88)	<0.001	3.46	(2.81–4.26)	<0.001	1.30	(1.22–1.39)	<0.001
	Model 2	2.23	(2.11–2.35)	<0.001	1.72	(1.61–1.83)	<0.001	2.13	(2.05–2.22)	<0.001	3.27	(2.64–4.05)	<0.001	1.25	(1.17–1.33)	<0.001
	Model 3	1.71	(1.61–1.82)	<0.001	1.37	(1.28–1.46)	<0.001	1.70	(1.63–1.78)	<0.001	2.59	(2.06–3.25)	<0.001	1.17	(1.09–1.25)	<0.001
	Model 4	1.54	(1.45–1.64)	<0.001	1.25	(1.17–1.33)	<0.001	1.43	(1.37–1.49)	<0.001	2.45	(1.95–3.09)	<0.001	1.07	(1.00–1.14)	0.040

BADL: basic activities of daily living; CHARLS: China Health and Retirement Longitudinal Study; CI: confidence interval; ELSA: English Longitudinal Study of Ageing; HRS: Health and Retirement Study; IADL: instrumental activities of daily living; IRR: incidence rate ratio; MHAS: Mexican Health and Aging Study; SHARE: Survey of Health, Ageing and Retirement in Europe.

Model 1 was crude model.

Model 2 was adjusted for gender and age.

Model 3 was adjusted for the minimal sufficient adjustment set (MSAS) identified using a causal directed acyclic graph (DAG) including further adjusted for labour force status, education, household wealth, marital status, and co-residence with children based on Model 2.

Model 4 was further adjusted for smoking, drinking, ever had hypertension, ever had stroke, ever had cancer, depressive symptoms, and cognitive impairment based on Model 3.

**Table 4:** Association between digital exclusion and functional dependency.

	HRS			ELSA			SHARE			CHARLS			MHAS		
	RRR	95% CI	*p* value	RRR	95% CI	*p* value	RRR	95% CI	*p* value	RRR	95% CI	*p* value	RRR	95% CI	*p* value
Independent	Ref			Ref			Ref			Ref			Ref		
Low dependency	1.71	(1.41–2.08)	<0.001	1.04	(0.83–1.31)	0.734	1.18	(0.97–1.43)	0.098	1.21	(0.57–2.56)	0.621	0.64	(0.52–0.80)	<0.001
Medium dependency	1.14	(0.77–1.68)	0.510	2.08	(0.69–6.23)	0.191	1.02	(0.48–2.19)	0.952	0.80	(0.12–5.37)	0.816	0.81	(0.50–1.32)	0.391
High dependency	1.26	(1.02–1.55)	0.029	0.97	(0.69–1.38)	0.874	0.86	(0.63–1.16)	0.319	2.19	(0.77–6.26)	0.142	0.93	(0.77–1.14)	0.491

CHARLS: China Health and Retirement Longitudinal Study; CI: confidence interval; ELSA: English Longitudinal Study of Ageing; HRS: Health and Retirement Study; MHAS: Mexican Health and Aging Study; RRR: relative-risk ratio; SHARE: Survey of Health, Ageing and Retirement in Europe.

Models were adjusted for the minimal sufficient adjustment set (MSAS) identified using a causal directed acyclic graph (DAG) including gender, age, education, labour force status, marital status, household wealth, and co-residence with children.

In sensitivity analyses, we repeated the GEE model controlling MSAS by treating the outcomes as continuous variables (scores of BADL and IADL), and the associations remained statistically significant among all cohort studies (Supplementary Table S3). In the follow-up cohort study, the incidence rate of older adults with difficulties in BADL or IADL was higher in those excluded from the internet compared to their counterparts in all cohorts except for those in MHAS, who showed the opposite results, that the incidence rate was lower in those excluded from the internet (Supplementary Table S4). After excluding participants with severe cognitive impairment at baseline or using IPW, the analyses in Table 3 have been repeated and the associations remained (Supplementary Table S5 and S6).

**Fig. 2:**
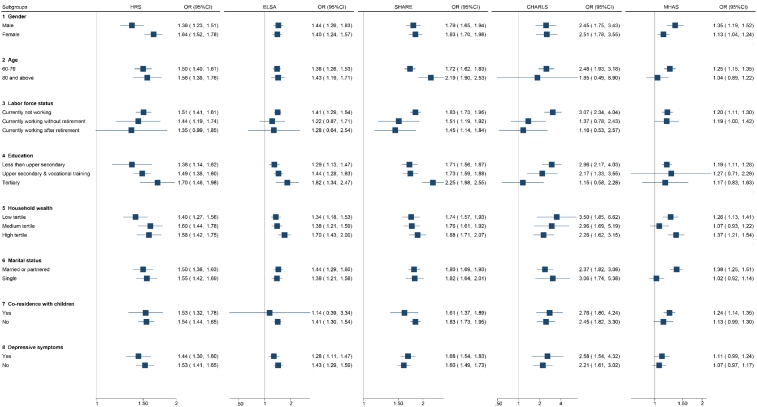
Association between digital exclusion and BADL by gender, age, labour force status, education, household wealth, marital status, co-residence with children, and depressive symptoms.

**Fig. 3:**
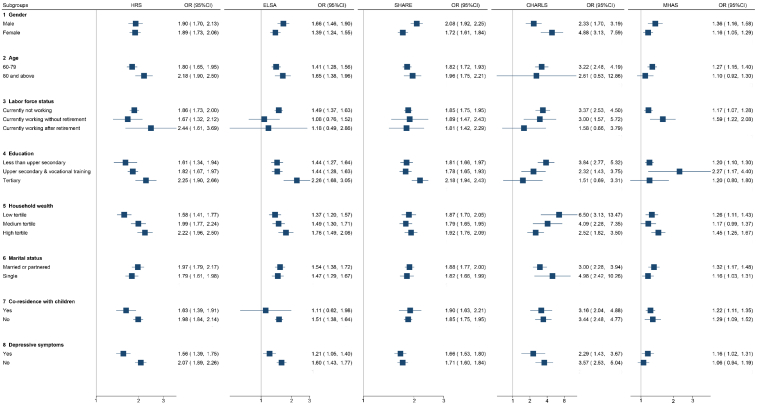
Association between digital exclusion and IADL by gender, age, labour force status, education, household wealth, marital status, co-residence with children, and depressive symptoms.

To assess the heterogeneity of digital exclusion on BADL and IADL, Figs. 2 and 3 visualize the digital exclusion-ADL association in different subpopulations. In general, we found a stronger negative association between digital exclusion and BADL or IADL in adults aged 80 and above, currently not working, not co-resident with children, and without depressive symptoms.

## Discussion

For older people aged 60 and above from our harmonised cohorts, digital exclusion rates range from 23.8% in Denmark and 30.4% in England to 65.5% in Mexico and 96.9% in China. The results of our study were consistent with the finding that HICs were more digitalised than middle-income countries (MICs). We also reported that the incidence rate of functional dependency in those excluded from digital was higher compared to those included by digital in both HICs (HRS, ELSA, SHARE) and MICs (CHARLS, MHAS). The associations were stronger in the older population.

To the best of our knowledge, this study was the first to examine the longitudinal association between digital exclusion and functional dependency in terms of BADL and IADL among the older population. Although prior studies described the digital divide in people with physical and mental impairment,^22^, ^23^, ^24^, ^25^ there is less evidence on the link of digital exclusion on disability and functional dependency. With the exponentially growing proportion of the population connected to the internet over the past decade, researchers in the field of health functioning improvement have been quick to capitalize on the internet to promote health management in many settings.^26^^,^^27^ However, the rate of digital exclusion in the older population remains relatively high, especially in MICs such as China, where economic growth and population aging are happening simultaneously, limiting the potential of the internet as a platform for achieving better health management. Taking advantage of the multi-country population-based cohorts, we were able to investigate whether the effect of digital exclusion on functional dependency differed in HICs and LMICs, and more importantly, what groups of the older population are more sensitive to digital exclusion in terms of functional dependency.

By including five cohorts from 23 countries on three continents, digital exclusion was found to be associated with difficulty in ADL and functional dependency in the older population. The finding paralleled that of previous studies proving that adults using the internet for e-mail or online messages had a significantly lower risk of physical limitations.^4^^,^^28^, ^29^, ^30^, ^31^ However, few have investigated whether this effect still holds in a developing context. The present study found the association was more notable and statistically significant in MICs with a higher proportion of digital exclusion, such as China. One possible explanation is that the marginal effect of digital exclusion was decreasing as the coverage of internet use was increasing. This suggests the urgency and significance of addressing the digital exclusion issues among the older population to slow their decline in physical functions in MICs. The findings are also practical in the sense that MICs are facing relatively great health challenges of aging and functional dependency in which health promotion and changes in health behaviour are the necessary steps to health management for relieving functional impairment. More broadly, the present findings suggest that bridging the digital divide in an aging society could be of help in decreasing the health disparity in functional dependency between HICs and LMICs.

We explored the potential mechanisms for explaining the association between digital exclusion and functional dependency. First, digital exclusion was associated with every item of BADL or IADL, which suggested it to be a potential protective factor to improve the overall health functioning of the older population. Via the internet, the elderly population can get access to and follow the latest information in health management and purchase medication and health devices.^32^ The internet also provides the older population opportunities for timely consulting with health care professionals as well as real-time data monitoring.^33^^,^^34^ Second, vulnerable digital excluders, such as those who had a lower level of social communication with colleagues or family members, were at higher odds of functional dependency, compared to those who were not in a vulnerable position. This is in line with a series of studies emphasizing the fact that internet users participate in more social activities,^35^ alleviate social loneliness owing to physical activities,^36^^,^^37^ and engage in beneficial health behaviors.^4^^,^^37^ It seems that the internet has become an alternative pathway to real–life interaction to alleviate social isolation and improve the quality of life for older adults.^38^^,^^39^ Third, the active involvement in the internet among older adults implied that they were competent in self-management^40^ and thus qualified for daily activities. What cannot be ignored is the initiative of older adults with disabilities, who may be empowered to grasp the rich opportunities of the internet to address their health concerns.

The present findings implicate that access to the internet can decrease the likelihood of functioning dependency in the elderly, especially in vulnerable populations, which highlightes that a tailored digital inclusion strategy could promote an active ageing process. In the continuing spread of the COVID-19 pandemics, given the inconvenience of face-to-face interaction, the older population with functional dependence was more susceptible to being exposed to depression and loneliness.^41^ It has been well-documented that the internet provides a substitute way of reaching out to society and promoting access to health care.^42^, ^43^, ^44^ Positive empirical evidence has accumulated in the field of health management, indicating that e-health services have successfully emerged as a useful complement to health care and a vital part of an inclusive health care system.^45^ Building up a society of digital inclusion in the aging process helps people access early, timely, and long-term health management in their later lives. Recent studies have piloted internet-based interventions to cope with the functional dependency of the aged. For example, one randomized controlled trial proved that ICT training and smartphone technology utilization reduce difficulties in IADL.^46^^,^^47^ An internet-based health education tool was verified to be effective in improving disability with low cost, thereby reducing the health care burden of older adults.^48^ Nonetheless, more robust evidence of the positive effects of digital inclusion in the older population is warranted, and interventional trials should be expanded to more countries and regions to identify tailored strategies to bridge the digital divide.

Our study has several strengths. First, the results of the cross-cultural, longitudinal study demonstrate the generalisation since the sample consisted of five individual-level cohorts from 23 countries, both HICs and LMICs, across three continents. Second, all participants were recruited from large national representative samples, and the five surveys were standardized for cross-database comparisons. Third, GEE models that take into account correlations among multiple waves of longitudinal data reduce the likelihood of misestimation.

Certain limitations should be acknowledged. First, there is information bias due to different measurements of exposure, which use internet use at an individual level in HRS, ELSA, SHARE, and CHARLS and internet access at a household level in MHAS. Second, the extent to which the participants use the internet was not considered due to a lack of data availability. Third, we categorized confounders such as age and household wealth, which can result in residual confounding. Fourth, there are unmeasured covariates in the present study, including social interaction and communication, community support conditions, and other time-varying confounding. Fifth, we could not exclude the possibility of reverse association between digital exclusion and functional dependence, despite the Poisson model calculating IRR to gain more accurate estimations. In fact, previous studies focused on the moderating effects of functional disability on internet use in older adults.^24^^,^^49^ Further experimental studies are needed in order to infer a causal link between digital inclusion strategy and the burden of functional dependency.

## Contributors

Yinzi Jin and Yao Yao conceptualized study design. Xinran Lu and Yao Yao conducted investigation and methodology. Xinran Lu implemented data curation, statistical analysis, and drafted the manuscript. Yao Yao verified the analysis. Yinzi Jin and Yao Yao reviewed and edited the manuscript. Yinzi Jin contributed to the funding acquisition and supervised the research. All authors had full access to the data and accept the responsibility to submit the manuscript.

## Data sharing statement

Original survey datasets from HRS, ELSA, SHARE, CHARLS, and MHAS are freely available to all bona fide researchers. The data that support the findings of this study are available from the GATEWAY TO GLOBAL AGING DATA (https://g2aging.org/).

## Declaration of interests

The authors declare no competing interests.

## Appendix A Supplementary data

Supplementary Figures A1, A2 and Tables A1–A6
