# Corrigendum to “Digital exclusion and functional dependence in older people: findings from five longitudinal cohort studies” eClinical Medicine 54 (2022) 101708

**DOI:** 10.1016/j.eclinm.2023.102239

**Published:** 2023-10-02

**Authors:** Xinran Lu, Yao Yao, Yinzi Jin

**Affiliations:** a Department of Global Health, School of Public Health, Peking University, Beijing, China; b China Center for Health Development Studies, Peking University, Beijing, China; c Institute for Global Health and Development, Peking University, Beijing, China

In the original published version of this article, the author has identified some errors in the classification of dependency. They have made the corrections and re-analysis the data, and the results and conclusions continued to be consistent.

The code in the published version does not accurately represent the definition of functional dependencies (Table 1). Specifically, "low dependency" is defined as "difficulty in bathing, managing money, shopping for groceries, using the telephone, or cleaning the house, as well as no difficulty in any of the items defined as medium and high dependency". Inadvertently, the analysis failed to exclude individuals who did not report difficulties with the items covered by medium and high dependency criteria. Also, there is a discrepancy in the definition of "Medium dependency." The analysis did not account for participants who did not display difficulties in items of high dependency. As a result, the proportion of high dependency cases is currently lower than anticipated.

**Table 1:** Functional dependency by interval-of-need dependency categorization.

Categories	Definition
High dependency	Difficulty in eating, dressing, getting in/out of bed, using the toilet, or walking
Medium dependency	Difficulty in preparing hot meals or taking medications, and no-difficulty in the items defined in high dependency
Low dependency	Difficulty in bathing, managing money, shopping for groceries, using the telephone, or cleaning the house, and no-difficulty in the items defined in medium and high dependency
Independent	No-difficulty in the items above

**Revised Table 2:** Descriptive statistics in HRS, ELSA, SHARE, CHARLS, and MHAS.

	HRS (N = 49,583)	ELSA (N = 27,338)	SHARE (N = 96,184)	CHARLS (N = 23,342)	MHAS^a^ (N = 26,968)
Age, median (Q1–Q3)	72 (65–78)	69 (64–76)	70 (12–77)	67 (63–72)	69 (65–76)
Male gender	20,469 (41.3)	12,853 (47.0)	42,160 (43.8)	11,261 (48.2)	11,854 (44.0)
Labour force status					
Currently not working	36,479 (73.6)	21,670 (79.3)	78,390 (81.5)	11,214 (48.0)	19,162 (71.1)
Currently working without retirement	10,404 (21.0)	4846 (17.7)	10,737 (11.2)	11,095 (47.5)	7806 (28.9)
Currently working after retirement	2700 (5.45)	822 (3.01)	7057 (7.34)	1033 (4.43)	
Education					
Less than upper secondary	9381 (18.9)	8003 (29.3)	42,587 (44.3)	21,649 (92.7)	23,949 (88.8)
Upper secondary and vocational training	28,989 (58.5)	13,955 (51.0)	33,529 (34.9)	1363 (5.84)	691 (2.56)
Tertiary	11,213 (22.6)	5380 (19.7)	20,068 (20.9)	330 (1.41)	2328 (8.63)
Household wealth					
Low tertile	14,998 (30.2)	7464 (27.3)	28,993 (30.1)	7253 (31.1)	10,825 (40.1)
Medium tertile	14,974 (30.2)	9423 (34.5)	33,028 (34.3)	9231 (39.5)	7322 (27.2)
High tertile	19,611 (39.6)	10,451 (38.2)	34,163 (35.5)	6858 (29.4)	8821 (32.7)
Married or partnered	29,735 (60.0)	19,171 (70.1)	69,294 (72.0)	18,341 (78.6)	17,000 (63.0)
Co-residence with children	7908 (15.9)	224 (0.82)	13,692 (14.2)	9270 (39.7)	18,302 (67.9)
Smoking	5181 (10.4)	2462 (9.01)	13,667 (14.2)	6294 (27.0)	2689 (9.97)
Alcohol drinking	25,587 (51.6)	23,557 (86.2)	47,555 (49.4)	7245 (31.0)	5905 (21.9)
Ever had hypertension	32,657 (65.9)	12,854 (47.0)	52,886 (55.0)	9456 (40.5)	16,907 (62.7)
Ever had stroke	4957 (10.0)	1341 (4.91)	7320 (7.61)	1365 (5.85)	1192 (4.42)
Ever had cancer	8946 (18.0)	3621 (11.9)	10,595 (11.0)	378 (1.62)	1101 (4.08)
Depressive symptom	10,314 (20.8)	5057 (18.5)	26,091 (27.1)	8931 (38.3)	8760 (32.5)
Cognitive impairment	2275 (4.59)	1047 (3.83)	5117 (5.32)	327 (1.40)	1652 (6.13)
Digital exclusion	26,394 (53.2)	8316 (30.4)	55,201 (57.4)	22,622 (96.9)	17,653 (65.5)
BADL, median (Q1–Q3)	0 (0–0)	0 (0–0)	0 (0–0)	0 (0–1)	0 (0–0)
IADL, median (Q1–Q3)	0 (0–0)	0 (0–0)	0 (0–0)	0 (0–1)	0 (0–0)
Difficulty in BADL	0.42 (1.07)	0.33 (0.90)	0.26 (0.85)	0.54 (1.16)	0.45 (1.10)
Difficulty in IADL	0.30 (0.82)	0.31 (0.81)	0.33 (0.95)	0.72 (1.29)	0.26 (0.73)
Functional dependency					
Independent	38,780 (71.2)	26,429 (72.7)	85,775 (77.6)	20,843 (57.0)	20,666 (70.7)
Low dependency	2674 (4.9)	3002 (8.3)	8886 (8.0)	4201 (11.5)	919 (3.2)
Medium dependency	1639 (3.0)	577 (1.6)	2208 (2.0)	2191 (6.0)	568 (1.9)
High dependency	11,412 (20.9)	6351 (17.5)	13,655 (12.4)	9358 (25.6)	7066 (24.2)

BADL: basic activities of daily living; CHARLS: China Health and Retirement Longitudinal Study; ELSA: English Longitudinal Study of Ageing; HRS: Health and Retirement Study; IADL: instrumental activities of daily living; MHAS: Mexican Health and Aging Study; SHARE: Survey of Health, Ageing and Retirement in Europe.

Data are N (%) for categorical variables or mean (SD) or median (Q1–Q3) for continuous variables.

a For MHAS, the question on retirement was unavailable, so labour force status was recoded into currently working and currently not working.

**Revised Fig. 1:**
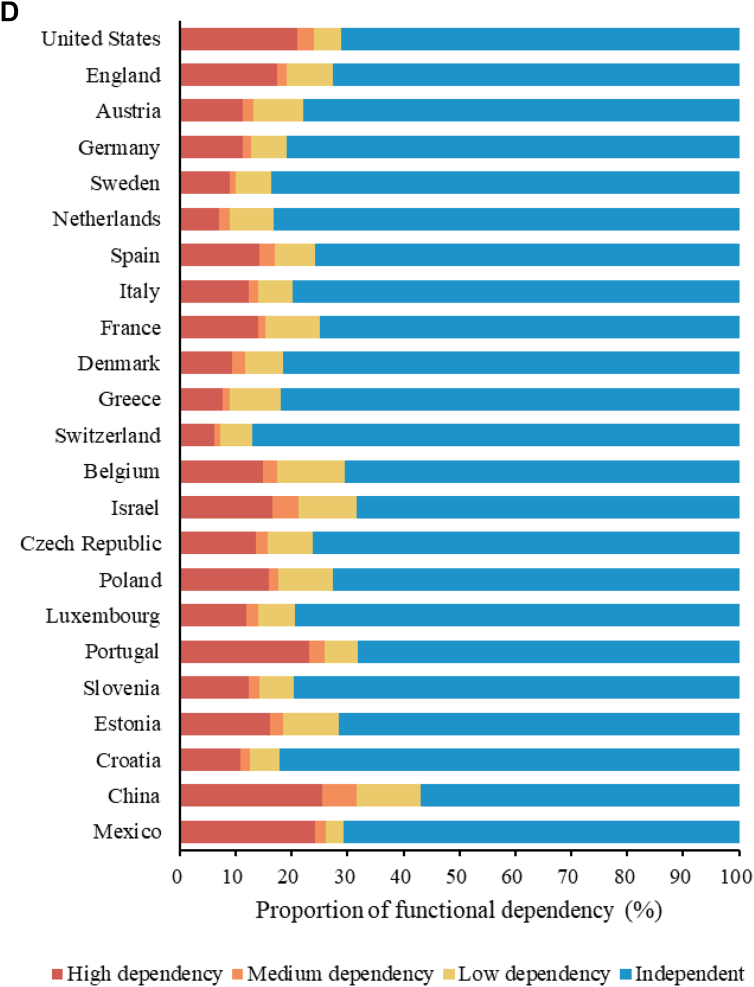
Proportion of digital exclusion, difficulties in BADL, difficulties in IADL, and functional dependency.

Below, we take part of the Stata code for example to show the differences of generation of functional dependency variable. The variable *r`wv'dependency* was defined as functional dependency, where the values are categorized as follows: 0 for independence, 1 for low dependency, 2 for medium dependency, and 3 for high dependency. Variables including *r`wv'dressa*, *r`wv'eata*, *r`wv'beda*, *r`wv'toilta*, *r`wv'walk100a*, *r`wv'medsa*, *r`wv'mealsa*, *r`wv'moneya*, *r`wv'shopa*, *r`wv'housewka*, *r`wv'phonea*, *r`wv'batha* refers to items contributing to the determination of functional dependency. *r`wv'adl* and *r`wv'iadl* represent the count of difficulties in items related to activities of daily living (ADL) and instrumental activities of daily living (IADL), respectively. ADL and IADL variables include all the above variables contributing to the definition of functional dependency.

Original Stata code

*forvalues wv = 2/4{*

*gen r`wv'dependency = .*

*replace r`wv'dependency = 3 if (r`wv'dressa == 1 | r`wv'eata == 1 | r`wv'beda == 1 | r`wv'toilta == 1 | r`wv'walk100a == 1)*

*replace r`wv'dependency = 2 if (r`wv'medsa == 1 | r`wv'mealsa == 1)*

*replace r`wv'dependency = 1 if (r`wv'moneya == 1 | r`wv'shopa == 1 | r`wv'housewka == 1 | r`wv'phonea == 1 | r`wv'batha == 1)*

*replace r`wv'dependency = 0 if (r`wv'adl == 0 & r`wv'iadl == 0)*

*}*

Revised Stata code

*forvalues wv = 2/4{*

*gen r`wv'dependency = .*

*replace r`wv'dependency = 0 if (r`wv'adl == 0 & r`wv'iadl == 0)*

*replace r`wv'dependency = 1 if (r`wv'moneya == 1 | r`wv'shopa == 1 | r`wv'housewka == 1 | r`wv'phonea == 1 | r`wv'batha == 1)*

*replace r`wv'dependency = 2 if (r`wv'medsa == 1 | r`wv'mealsa == 1)*

*replace r`wv'dependency = 3 if (r`wv'dressa == 1 | r`wv'eata == 1 | r`wv'beda == 1 | r`wv'toilta == 1 | r`wv'walk100a == 1)*

*}*

Adjustments were made to the relevant results, including one figure and two tables in the main text, as well as one table in the appendix.

In general, there has been an increase in the proportion of individuals with high dependency while the proportions of other functional statuses have shown relative changes. In Table 4, we now observe the statistical significance of medium dependency in Health and Retirement Study (HRS) and high dependency in Mexican Health and Aging Study (MHAS).

**Revised Table 4:** Association between digital exclusion and functional dependency.

	HRS			ELSA			SHARE			CHARLS			MHAS		
	RRR	95% CI	*p* value	RRR	95% CI	*p* value	RRR	95% CI	*p* value	RRR	95% CI	*p* value	RRR	95% CI	*p* value
Independent	Ref			Ref			Ref			Ref			Ref		
Low dependency	1.59	(1.25–2.04)	<0.001	0.97	(0.75–1.26)	0.812	1.06	(0.84–1.33)	0.639	1.31	(0.52–3.35)	0.567	0.78	(0.57–1.08)	0.131
Medium dependency	1.85	(1.28–2.66)	0.001	1.52	(0.79–2.93)	0.212	1.19	(0.69–2.06)	0.527	1.14	(0.38–3.42)	0.820	0.73	(0.47–1.12)	0.151
High dependency	1.33	(1.11–1.59)	0.002	1.05	(0.81–1.36)	0.717	1.09	(0.87–1.37)	0.461	1.58	(0.69–3.58)	0.277	0.80	(0.68–0.95)	0.009

CHARLS: China Health and Retirement Longitudinal Study; CI: confidence interval; ELSA: English Longitudinal Study of Ageing; HRS: Health and Retirement Study; MHAS: Mexican Health and Aging Study; RRR: relative-risk ratio; SHARE: Survey of Health, Ageing and Retirement in Europe.

Models were adjusted for the minimal sufficient adjustment set (MSAS) identified using a causal directed acyclic graph (DAG) including gender, age, education, labour force status, marital status, household wealth, and co-residence with children.

**Revised Table A1:** Distribution of digital exclusion, difficulties in BADL/IADL, and functional dependency of study participants by geographic regions.

	Digital exclusion	Difficulty in BADL	Difficulty in IADL	Functional dependency			
				Independent	Low dependency	Medium dependency	High dependency
HRS							
United States	53.2	19.6	16.3	71.2	4.9	3.0	20.9
ELSA							
England	35.1	17.5	17.3	72.7	8.3	1.6	17.5
SHARE							
Austria	59.3	11.2	17.0	77.9	8.9	1.9	11.2
Germany	52.1	11.7	13.6	80.8	6.5	1.5	11.3
Sweden	28.6	8.5	10.9	83.7	6.2	1.2	8.9
Netherlands	30.5	7.0	12.6	83.3	7.8	1.8	7.2
Spain	79.9	13.0	17.2	75.7	7.3	2.8	14.2
Italy	79.5	12.3	13.7	79.7	6.3	1.6	12.5
France	51.3	14.4	17.7	74.9	9.8	1.4	14.0
Denmark	23.8	8.5	13.1	81.5	6.9	2.3	9.4
Greece	83.0	8.9	16.0	81.9	9.1	1.2	7.8
Switzerland	37.9	6.7	8.7	87.0	5.7	1.1	6.2
Belgium	47.1	16.8	20.9	70.4	12.0	2.5	15.0
Israel	52.9	13.9	25.0	68.3	10.4	4.7	16.6
Czech Republic	60.7	13.7	16.6	76.2	8.1	2.1	13.6
Poland	84.6	17.4	24.1	72.6	9.7	1.7	16.0
Luxembourg	49.5	11.0	15.1	79.4	6.6	2.1	11.9
Portugal	82.2	25.5	21.2	68.2	6.0	2.7	23.1
Slovenia	72.1	11.9	14.8	79.5	6.2	2.0	12.3
Estonia	67.4	17.8	21.6	71.5	10.0	2.4	16.1
Croatia	80.0	11.5	12.2	82.1	5.3	1.7	11.0
CHARLS							
China	96.9	25.7	33.1	57.0	11.5	6.0	25.6
MHAS							
Mexico	65.5	21.5	15.0	70.7	3.2	1.9	24.2

BADL: basic activities of daily living; CHARLS: China Health and Retirement Longitudinal Study; ELSA: English Longitudinal Study of Ageing; HRS: Health and Retirement Study; IADL: instrumental activities of daily living; MHAS: Mexican Health and Aging Study; SHARE: Survey of Health, Ageing and Retirement in Europe.

