# Supplementary material for: Digital exclusion and functional dependence in older people: Findings from five longitudinal cohort studies
Source: eClinicalMedicine. 2022 Oct 31;54:101708. doi: 10.1016/j.eclinm.2022.101708 (PMC9637559; doi:10.1016/j.eclinm.2022.101708)
Supplement: Supplementary Figures A1, A2 and Tables A1–A6 [file mmc1.pdf]

Supplementary Information

Digital exclusion and Functional dependence in older people: findings from five longitudinal cohort studies

Xinran Lu, Yao Yao, Yinzi Jin

Contents

Figure A1. Study flow diagrams 2
Figure A2. Causal directed acyclic graph 3
Supplementary Methods: Descriptions of covariates 4
Table A1. Distribution of digital exclusion, difficulties in BADL/IADL, and functional dependency of study participants by geographic regions. 5
Table A2. Association between digital exclusion and difficulties in each item of BADL and IADL 6
Table A3. Association between digital exclusion and scores of BADL and IADL. 7
Table A4. Sensitivity analyses of association between digital exclusion and difficulties in BADL and IADL in follow-up participants with non-difficulty in BADL and IADL at baseline. 8
Table A5. Sensitivity analyses of association between digital exclusion and difficulties in BADL and IADL by excluding participants with severe cognitive impairment at baseline. 9
Table A6. Sensitivity analyses of association between digital exclusion and difficulties in BADL and IADL in imputed datasets using inverse-probability weights. 10
Appendix References 11

Figure A1. Study flow diagrams

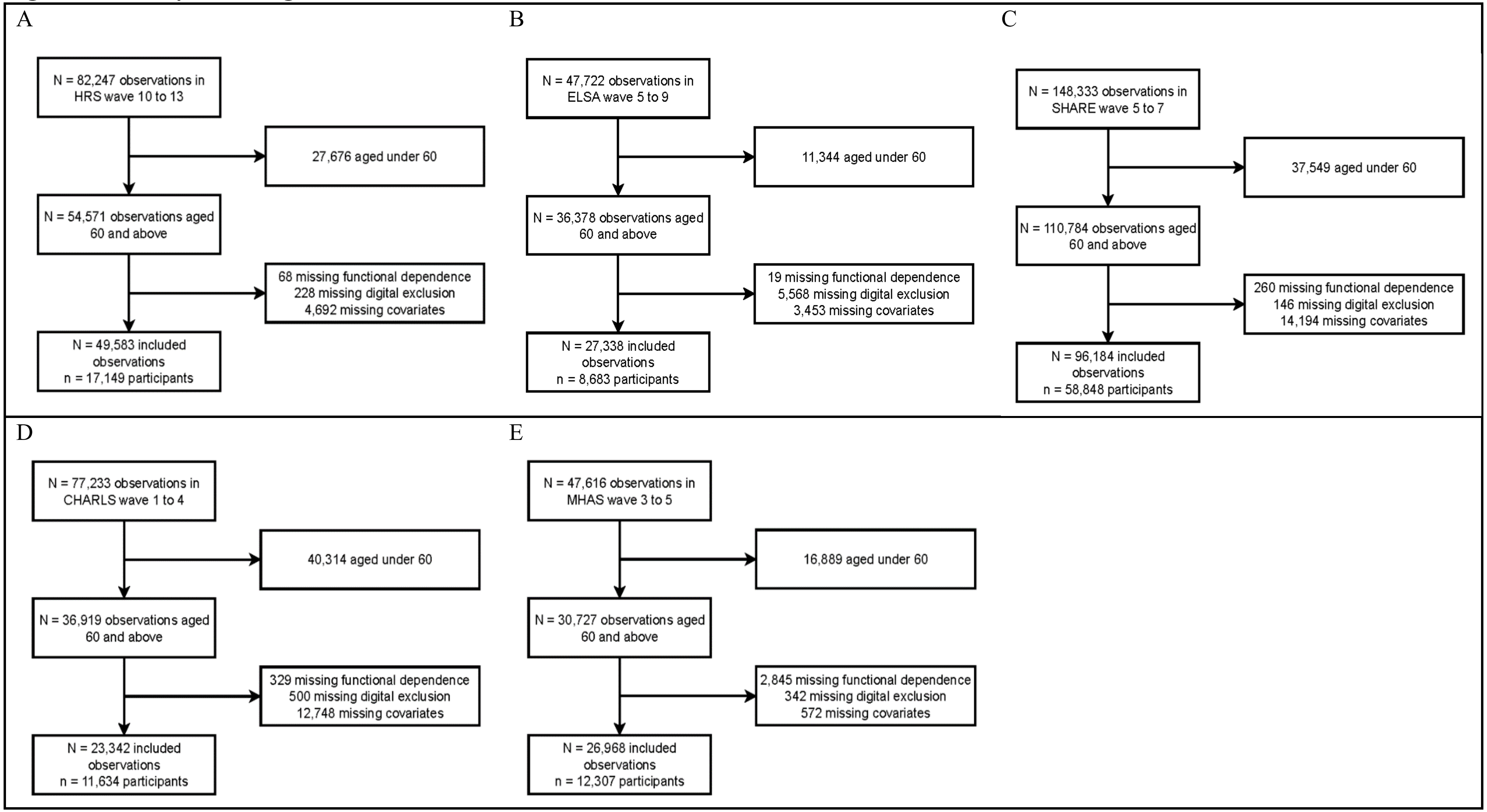

Note: HRS: Health and Retirement Study; ELSA: English Longitudinal Study of Ageing; SHARE: Survey of Health, Ageing and Retirement in Europe; CHARLS: China Health and Retirement Longitudinal Study; MHAS: Mexican Health and Aging Study.

Figure A2. Causal directed acyclic graph

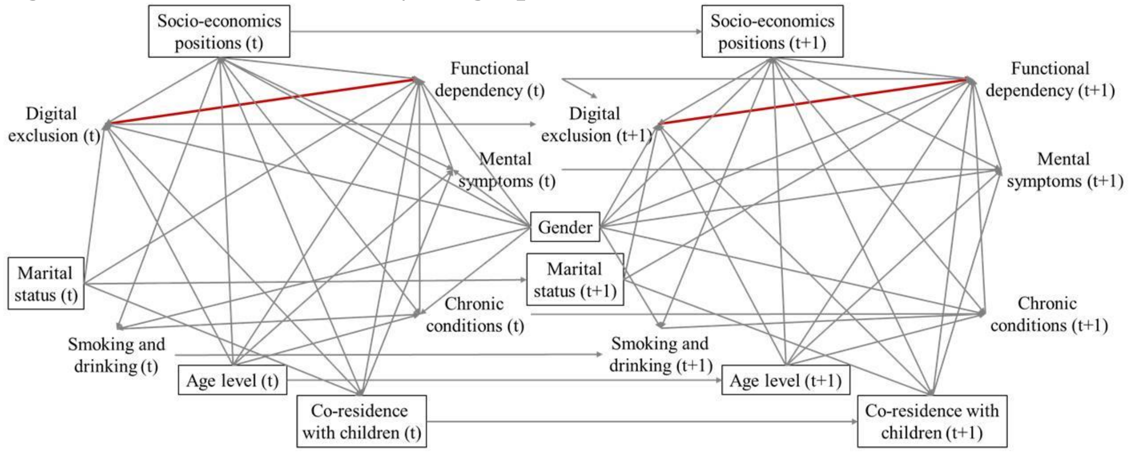

Note: The minimal sufficient adjustment set includes gender, age level, socio-economic positions (education, labour force status, and household wealth level), marital status, and co-residence with children, which were condition on in the present study. The red arrow shows the main effect of interest.

Supplementary Methods: Descriptions of covariates

The gender was reported as either male or female. At the time of the interview, age was recorded and classified as younger-old (60 to 79) and older-old (80 and over). According to the International Standard Classification of Education (ISCED) 1997, education is classified as lower secondary, upper secondary and vocational training, and tertiary. Using questions about current working status (working for pay or self-employed) and self-reported retirement, the labour force status was coded into currently not working, currently working without retirement, and currently working after retirement. The level of household wealth has been divided into tertiles of non-housing financial wealth. Marital status was categorised as married/partnered or single, single covered separated, divorced, widowed, and never married. Co-residence was viewed as a binary question, and no children missing was viewed as no co-residence. Smoking is described as current smoking behavior, while drinking is about whether alcohol was consumed last week, last year, or earlier.

Chronic conditions (hypertension, stroke, or cancer) are defined by whether or not the respondent had been advised by a doctor that they have or are currently suffering from these conditions. We measured depressive symptoms using the Centre for Epidemiologic Studies of Depression (CES-D) scale in CHARLS (CESD-10), HRS (CESD-8), ELSA (CESD-8), and MHAS (CESD-9), and Euro-D in SHARE with a score range of 0-8, 0-8, 0-12, 0-30, and 0-9, respectively. Participants with a scores equal to or greater than the cutoff score (HRS≥3, ELSA≥3, SHARE≥4, CHARLS≥10, and MHAS≥5) was coded as 1, others were coded as 0.[1-4] Three sets of cognitive function tests were completed to assess cognitive impairment, including an orientation to date test, an immediate word recall test, and a delayed word recall test. At orientation to date, participants were asked if they could remember the date of that day (day of week, day of month, month, and year in HRS, ELSA, SHARE, and SHARE; day of month, month, and year in MHAS). For the immediate and delayed word recall, participants were asked to recite 10 words in HRS, ELSA, SHARE, and SHARE; and 8 words in MHAS. Participants who performed 1.5 standard deviations below the mean of the score in two or three tests, compared to the total population aged 60 and over with the same level of education within the database were coded as 1, and other participants were coded as 0.[5-7]

Table A1. Distribution of digital exclusion, difficulties in BADL/IADL, and functional dependency of study participants by geographic regions.

		Digital exclusion	Difficulty in BADL	Difficulty in IADL	Functional dependency			
					Independent	Low dependency	Medium dependency	High dependency
**HRS**	United States	53.2	19.6	16.3	74.4	16.6	1.7	7.3
**ELSA**	England	35.1	17.5	17.3	76.1	18.9	0.3	4.7
**SHARE**	Austria	59.3	11.2	17.0	79.9	17.2	0.4	2.5
	Germany	52.1	11.7	13.6	82.3	14.3	0.3	3.1
	Sweden	28.6	8.5	10.9	85.4	11.0	0.3	3.3
	Netherlands	30.5	7.0	12.6	85.1	12.6	0.5	1.9
	Spain	79.9	13.0	17.2	79.6	17.8	0.3	2.3
	Italy	79.5	12.3	13.7	82.2	14.6	0.3	2.9
	France	51.3	14.4	17.7	76.8	18.6	0.2	4.4
	Denmark	23.8	8.5	13.1	84.0	12.9	0.6	2.6
	Greece	83.0	8.9	16.0	82.1	16.0	0.4	1.6
	Switzerland	37.9	6.7	8.7	88.0	9.0	0.4	2.6
	Belgium	47.1	16.8	20.9	73.3	22.6	0.5	3.6
	Israel	52.9	13.9	25.0	73.2	24.9	0.4	1.5
	Czech Republic	60.7	13.7	16.6	78.5	17.1	0.5	3.9
	Poland	84.6	17.4	24.1	71.2	25.1	0.1	3.6
	Luxembourg	49.5	11.0	15.1	82.0	14.9	0.7	2.4
	Portugal	82.2	25.5	21.2	67.5	22.0	0.5	10.1
	Slovenia	72.1	11.9	14.8	81.2	15.3	0.3	3.1
	Estonia	67.4	17.8	21.6	73.0	22.6	0.4	3.9
	Croatia	80.0	11.5	12.2	83.9	13.5	0.1	2.6
**CHARLS**	China	96.9	25.7	33.1	59.1	32.0	2.3	6.6
**MHAS**	Mexico	65.5	21.5	15.0	73.9	14.7	1.4	10.0

Note: BADL: basic activities of daily living; IADL: instrumental activities of daily living; HRS: Health and Retirement Study; ELSA: English Longitudinal Study of Ageing; SHARE: Survey of Health, Ageing and Retirement in Europe; CHARLS: China Health and Retirement Longitudinal Study; MHAS: Mexican Health and Aging Study.

Table A2. Association between digital exclusion and difficulties in each item of BADL and IADL.

	HRS			ELSA			SHARE			CHARLS			MHAS		
	IRR	95% CI	*p* value	IRR	95% CI	*p* value	IRR	95% CI	*p* value	IRR	95% CI	*p* value	IRR	95% CI	*p* value
**Eating**	2.18	(1.87 - 2.55)	<0.001	1.98	(1.53 - 2.55)	<0.001	2.39	(2.06 - 2.77)	<0.001	-†	-	-	1.15	(0.97 - 1.36)	0.106
**Dressing**	1.43	(1.33 - 1.53)	<0.001	1.26	(1.16 - 1.37)	<0.001	1.64	(1.54 - 1.74)	<0.001	2.77	(1.67 - 4.59)	<0.001	1.13	(1.05 - 1.22)	0.002
**Getting in/out of bed**	1.66	(1.51 - 1.84)	<0.001	1.32	(1.16 - 1.51)	<0.001	2.22	(2.00 - 2.45)	<0.001	4.15	(2.26 - 7.59)	<0.001	1.26	(1.15 - 1.39)	<0.001
**Using the toilet**	1.63	(1.47 - 1.81)	<0.001	1.24	(1.05 - 1.47)	0.014	2.26	(1.98 - 2.57)	<0.001	2.26	(1.69 - 3.04)	<0.001	1.22	(1.09 - 1.36)	<0.001
**Bathing**	1.82	(1.66 - 2.00)	<0.001	1.52	(1.37 - 1.69)	<0.001	2.43	(2.23 - 2.65)	<0.001	3.42	(2.15 - 5.46)	<0.001	1.17	(1.04 - 1.33)	0.010
**Walking**	1.59	(1.45 - 1.74)	<0.001	1.45	(1.20 - 1.75)	<0.001	2.79	(2.40 - 3.23)	<0.001	3.39	(2.04 - 5.63)	<0.001	1.16	(1.06 - 1.27)	0.002
**Preparing hot meals**	2.18	(1.96 - 2.41)	<0.001	1.76	(1.48 - 2.10)	<0.001	3.12	(2.79 - 3.50)	<0.001	3.55	(2.38 - 5.29)	<0.001	1.10	(0.99 - 1.23)	0.072
**Taking medications**	1.87	(1.59 - 2.19)	<0.001	2.50	(1.91 - 3.26)	<0.001	6.18	(4.99 - 7.65)	<0.001	3.14	(1.73 - 5.71)	<0.001	1.30	(1.12 - 1.50)	<0.001
**Managing money**	2.41	(2.15 - 2.70)	<0.001	2.83	(2.22 - 3.60)	<0.001	4.82	(4.23 - 5.48)	<0.001	4.91	(2.94 - 8.18)	<0.001	1.24	(1.07 - 1.44)	0.003
**Shopping for groceries**	1.68	(1.55 - 1.82)	<0.001	1.71	(1.53 - 1.92)	<0.001	2.64	(2.42 - 2.87)	<0.001	4.03	(2.46 - 6.62)	<0.001	1.14	(1.06 - 1.22)	<0.001
**Using the telephone**	3.40	(2.90 - 3.98)	<0.001	2.39	(1.86 - 3.06)	<0.001	3.47	(2.90 - 4.16)	<0.001	9.71	(4.97 - 18.99)	<0.001	-	-	-
**Cleaning the house**	-	-	-	1.29	(1.19 - 1.39)	<0.001	1.62	(1.54 - 1.70)	<0.001	2.41	(1.76 - 3.29)	<0.001	-	-	-

Note: BADL: basic activities of daily living; IADL: instrumental activities of daily living; OR: odds ratio; CI: confidence interval; HRS: Health and Retirement Study; ELSA: English Longitudinal Study of Ageing; SHARE: Survey of Health, Ageing and Retirement in Europe; CHARLS: China Health and Retirement Longitudinal Study; MHAS: Mexican Health and Aging Study.

Models were adjusted for the minimal sufficient adjustment set (MSAS) identified using a causal directed acyclic graph (DAG) including gender, age, education, labour force status, marital status, household wealth, and co-residence with children.

† Since no participants both having difficulty in eating and being able to use the internet, the OR could not be calculated in CHARLS.

Table A3. Association between digital exclusion and scores of BADL and IADL.

		HRS			ELSA			SHARE			CHARLS			MHAS		
		IRR	95% CI	*p* value	IRR	95% CI	*p* value	IRR	95% CI	*p* value	IRR	95% CI	*p* value	IRR	95% CI	*p* value
**BADL**	Model 1	1.37	(1.34 - 1.40)	<0.001	1.25	(1.22 - 1.29)	<0.001	1.30	(1.29 - 1.32)	<0.001	1.41	(1.37 - 1.47)	<0.001	1.11	(1.08 - 1.15)	<0.001
	Model 2	1.32	(1.29 - 1.35)	<0.001	1.22	(1.18 - 1.26)	<0.001	1.21	(1.20 - 1.23)	<0.001	1.36	(1.31 - 1.41)	<0.001	1.09	(1.06 - 1.12)	<0.001
	Model 3	1.17	(1.15 - 1.20)	<0.001	1.13	(1.09 - 1.16)	<0.001	1.14	(1.12 - 1.15)	<0.001	1.25	(1.20 - 1.31)	<0.001	1.06	(1.03 - 1.09)	<0.001
	Model 4	1.13	(1.10 - 1.15)	<0.001	1.08	(1.05 - 1.11)	<0.001	1.07	(1.05 - 1.08)	<0.001	1.21	(1.16 - 1.26)	<0.001	1.02	(0.99 - 1.05)	0.283
**IADL**	Model 1	1.34	(1.32 - 1.36)	<0.001	1.35	(1.31 - 1.39)	<0.001	1.46	(1.44 - 1.47)	<0.001	1.65	(1.58 - 1.72)	<0.001	1.07	(1.05 - 1.09)	<0.001
	Model 2	1.29	(1.27 - 1.31)	<0.001	1.28	(1.25 - 1.32)	<0.001	1.29	(1.28 - 1.31)	<0.001	1.56	(1.50 - 1.63)	<0.001	1.05	(1.03 - 1.07)	<0.001
	Model 3	1.17	(1.15 - 1.19)	<0.001	1.17	(1.14 - 1.21)	<0.001	1.19	(1.18 - 1.21)	<0.001	1.38	(1.32 - 1.46)	<0.001	1.03	(1.01 - 1.05)	0.003
	Model 4	1.13	(1.11 - 1.14)	<0.001	1.11	(1.08 - 1.14)	<0.001	1.09	(1.08 - 1.10)	<0.001	1.31	(1.25 - 1.38)	<0.001	1.00	(0.98 - 1.02)	0.732

Note: BADL: basic activities of daily living; IADL: instrumental activities of daily living; OR: odds ratio; CI: confidence interval; HRS: Health and Retirement Study; ELSA: English Longitudinal Study of Ageing; SHARE: Survey of Health, Ageing and Retirement in Europe; CHARLS: China Health and Retirement Longitudinal Study; MHAS: Mexican Health and Aging Study.

Model 1 was crude model.

Model 2 was adjusted for gender and age.

Model 3 was adjusted for the minimal sufficient adjustment set (MSAS) identified using a causal directed acyclic graph (DAG) including further adjusted for labour force status, education, household wealth, marital status, and co-residence with children based on Model 2.

Model 4 was further adjusted for ever had hypertension, ever had stroke, ever had cancer, depressive symptoms, and cognitive impairment based on Model 3.

Table A4. Sensitivity analyses of association between digital exclusion and difficulties in BADL and IADL in follow-up participants with non-difficulty in BADL and IADL at baseline.

		HRS			ELSA			SHARE			CHARLS			MHAS		
		IRR	95% CI	*p* value	IRR	95% CI	*p* value	IRR	95% CI	*p* value	IRR	95% CI	*p* value	IRR	95% CI	*p* value
**Difficulty in BADL**	Model 3	1.27	(1.16 - 1.40)	<0.001	1.19	(1.04 - 1.37)	0.011	1.46	(1.31 - 1.63)	<0.001	1.74	(1.25 - 2.42)	0.001	0.91	(0.83 - 1.00)	0.056
	Model 4	1.17	(1.06 - 1.28)	<0.001	1.11	(0.97 - 1.26)	0.144	1.29	(1.16 - 1.44)	<0.001	1.67	(1.19 - 2.33)	0.003	0.83	(0.75 - 0.91)	<0.001
**Difficulty in IADL**	Model 3	1.69	(1.51 - 1.89)	<0.001	1.33	(1.17 - 1.51)	<0.001	1.46	(1.33 - 1.61)	<0.001	2.33	(1.64 - 3.32)	<0.001	0.95	(0.84 - 1.07)	0.371
	Model 4	1.45	(1.30 - 1.63)	<0.001	1.19	(1.05 - 1.35)	0.007	1.25	(1.14 - 1.37)	<0.001	2.21	(1.55 - 3.15)	<0.001	0.84	(0.75 - 0.95)	0.006

Note: BADL: basic activities of daily living; IADL: instrumental activities of daily living; OR: odds ratio; CI: confidence interval; HRS: Health and Retirement Study; ELSA: English Longitudinal Study of Ageing; SHARE: Survey of Health, Ageing and Retirement in Europe; CHARLS: China Health and Retirement Longitudinal Study; MHAS: Mexican Health and Aging Study.

Model 3 was adjusted for the minimal sufficient adjustment set (MSAS) identified using a causal directed acyclic graph (DAG) including further adjusted for labour force status, education, household wealth, marital status, and co-residence with children based on Model 2.

Model 4 was further adjusted for ever had hypertension, ever had stroke, ever had cancer, depressive symptoms, and cognitive impairment based on Model 3.

Table A5. Sensitivity analyses of association between digital exclusion and difficulties in BADL and IADL by excluding participants with severe cognitive impairment at baseline.

	HRS			ELSA			SHARE			CHARLS			MHAS		
	IRR	95% CI	*p* value	IRR	95% CI	*p* value	IRR	95% CI	*p* value	IRR	95% CI	*p* value	IRR	95% CI	*p* value
Difficulty in BADL	1.39	(1.32 - 1.46)	<0.001	1.30	(1.21 - 1.39)	<0.001	1.68	(1.57 - 1.79)	<0.001	2.01	(1.58 - 2.55)	<0.001	1.14	(1.07 - 1.20)	<0.001
Difficulty in IADL	1.67	(1.57 - 1.78)	<0.001	1.35	(1.25 - 1.45)	<0.001	1.64	(1.55 - 1.74)	<0.001	2.55	(1.96 - 3.31)	<0.001	1.13	(1.06 - 1.22)	0.001

Note: BADL: basic activities of daily living; IADL: instrumental activities of daily living; OR: odds ratio; CI: confidence interval; HRS: Health and Retirement Study; ELSA: English Longitudinal Study of Ageing; SHARE: Survey of Health, Ageing and Retirement in Europe; CHARLS: China Health and Retirement Longitudinal Study; MHAS: Mexican Health and Aging Study.

Models were adjusted for the minimal sufficient adjustment set (MSAS) identified using a causal directed acyclic graph (DAG) including gender, age, education, labour force status, marital status, household wealth, and co-residence with children.

Table A6. Sensitivity analyses of association between digital exclusion and difficulties in BADL and IADL in imputed datasets using inverse-probability weights.

	HRS			ELSA			SHARE			CHARLS			MHAS		
	IRR	95% CI	*p* value	IRR	95% CI	*p* value	IRR	95% CI	*p* value	IRR	95% CI	*p* value	IRR	95% CI	*p* value
Difficulty in BADL	1.65	(1.55 - 1.76)	<0.001	1.39	(1.28 - 1.52)	<0.001	1.86	(1.76 - 1.97)	<0.001	2.53	(1.63 - 3.94)	<0.001	1.13	(1.07 - 1.20)	<0.001
Difficulty in IADL	2.24	(2.08 - 2.41)	<0.001	1.47	(1.34 - 1.60)	<0.001	1.84	(1.76 - 1.94)	<0.001	3.44	(2.08 - 5.72)	<0.001	1.19	(1.10 - 1.28)	<0.001

Note: BADL: basic activities of daily living; IADL: instrumental activities of daily living; OR: odds ratio; CI: confidence interval; HRS: Health and Retirement Study; ELSA: English Longitudinal Study of Ageing; SHARE: Survey of Health, Ageing and Retirement in Europe; CHARLS: China Health and Retirement Longitudinal Study; MHAS: Mexican Health and Aging Study.

Outcome of the datasets were imputed using the inverse-probability weights (IPWs) under the missing at random assumption. IPWs were calculated based on dropout models using gender, age, education, labour force status, marital status, household wealth, and co-residence with children as covariates.

Models were adjusted for the minimal sufficient adjustment set (MSAS) identified using a causal directed acyclic graph (DAG) including gender, age, education, labour force status, marital status, household wealth, and co-residence with children.

Appendix References

1. Andresen EM, Malmgren JA, Carter WB, Patrick DL. Screening for depression in well older adults: evaluation of a short form of the CES-D (Center for Epidemiologic Studies Depression Scale). *Am J Prev Med* 1994; **10**(2): 77-84.
2. Dewey M, Prince M, Börsch-Supan A, et al. First Results from the Survey of Health, Ageing and Retirement in Europe (2004-2007). Mannheim Research Institute for the Economics of Aging; 2005.
3. Torres JM, Wong R. Childhood poverty and depressive symptoms for older adults in Mexico: a life-course analysis. *J Cross Cult Gerontol* 2013; **28**(3): 317-37.
4. Aguilar-Navarro SG, Fuentes-Cantu A, Avila-Funes JA, Garcia-Mayo EJ. [Validity and reliability of the screening questionnaire for geriatric depression used in the Mexican Health and Age Study]. *Salud publica de Mexico* 2007; **49**(4): 256-62.
5. Luchetti M, Terracciano A, Aschwanden D, Lee JH, Stephan Y, Sutin AR. Loneliness is associated with risk of cognitive impairment in the Survey of Health, Ageing and Retirement in Europe. *Int J Geriatr Psychiatry* 2020; **35**(7): 794-801.
6. Ahmadi-Abhari S, Guzman-Castillo M, Bandosz P, et al. Temporal trend in dementia incidence since 2002 and projections for prevalence in England and Wales to 2040: modelling study. *BMJ* 2017; **358**: j2856.
7. Han FF, Wang HX, Wu JJ, Yao W, Hao CF, Pei JJ. Depressive symptoms and cognitive impairment: A 10-year follow-up study from the Survey of Health, Ageing and Retirement in Europe. *Eur Psychiatry* 2021; **64**(1): e55.
